# Disorder and Photogeneration Efficiency in Organic Semiconductors

**DOI:** 10.1021/acs.jpclett.3c02120

**Published:** 2023-08-28

**Authors:** Artem V. Toropin, Vladimir R. Nikitenko, Nikolai A. Korolev, Oleg V. Prezhdo

**Affiliations:** † National Research Nuclear University “MEPhI” (Moscow Engineering Physics Institute), Moscow 115409, Russia; ‡ Department of Chemistry, University of Southern California, Los Angeles 90089, California, United States

## Abstract

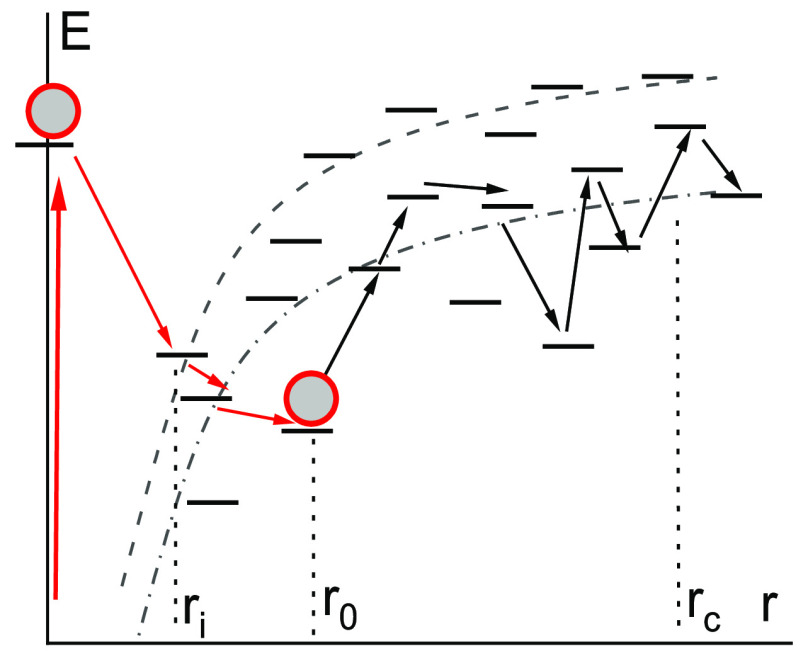

An analytical description of the separation probability of a geminate pair in organic semiconductors is given. The initial diffusion of “hot” twins is anomalously strong due to energy disorder. This circumstance significantly increases the photogeneration quantum yield at low temperatures and weakens its temperature dependence relative to predictions of the Onsager model, in agreement with Monte Carlo and experimental results.

Photogeneration and transport of charge carriers (electrons and holes) are the key physical processes that underlie the operation of photovoltaic devices based on disordered organic semiconductors.^1^ It is known that energy disorder determines the characteristics of hopping transport in these materials, in particular, the temperature and field dependence of the charge carrier mobility.^2^ The effect of disorder on the carrier photogeneration efficiency is undoubtedly significant. Usually, the problem is considered in the context of diffusion of molecular excitations (excitons), which is also controlled by energy disorder.^3^ However, in the case of fast exciton decay, the photogeneration efficiency is determined by the separation probability of an electron–hole pair bound by the Coulomb interaction (geminate pair).^4^ For the theoretical analysis of the geminate separation probability, modifications of the classical Onsager model^5,6^ are still mainly used, while they do not take into account the energy disorder inherent in organic semiconductors, as well as the hopping nature of the transport (discreteness of the medium).^4,7−9^ In addition, it is necessary to take into account the strongly nonequilibrium nature of the energy distribution of photogenerated carriers (“hot” carriers).^4^ At a sufficiently high energy of the exciting radiation, the initial energy relaxation of the “twins” occurs by hopping down in energy, so that their transport has an anomalous character.^10,11^ The effect of disorder and energy relaxation of carriers, which occurs during transport (pair separation), was modeled only by the Monte Carlo method.^12−14^ The above circumstances limit the applicability of the classical Onsager model and its modifications, especially at sufficiently low temperatures. Indeed, according to the experimental data, the photogeneration quantum yield depends on temperature much weaker than predicted by the Onsager model.^4,15^ The aim of this work is to develop a theoretical model of geminate pair separation that takes into account the hopping nature of transport and energy disorder and enables analytical modeling of the photogeneration efficiency, in addition to the Monte Carlo simulations.

The Onsager model assumes that a more mobile carrier (for definiteness, an electron) performs a diffusion-drift motion in the Coulomb field of a less mobile “twin”. In disordered inorganic semiconductors, the hot carrier thermalizes within a short initial time interval, after which it occupies a state near the mobility edge,^16^*Ec*. In organic materials, the energy *Ec* should be understood as the transport level.^17−19^ In these materials, the primary excitation is usually a molecular excitation (exciton); that is, an electron and a hole are on the same molecule. Further, it should be expected that the more mobile carrier jumps to one of the nearest molecular states, and a geminate pair (charge-transfer state) is formed.^4,7^ In this case, the distance between the “twin” charges is on the order of the average intermolecular distance, *r*_i_ ≅ *a* ≅ 1 nm, see Figure 1.

**Figure 1:**
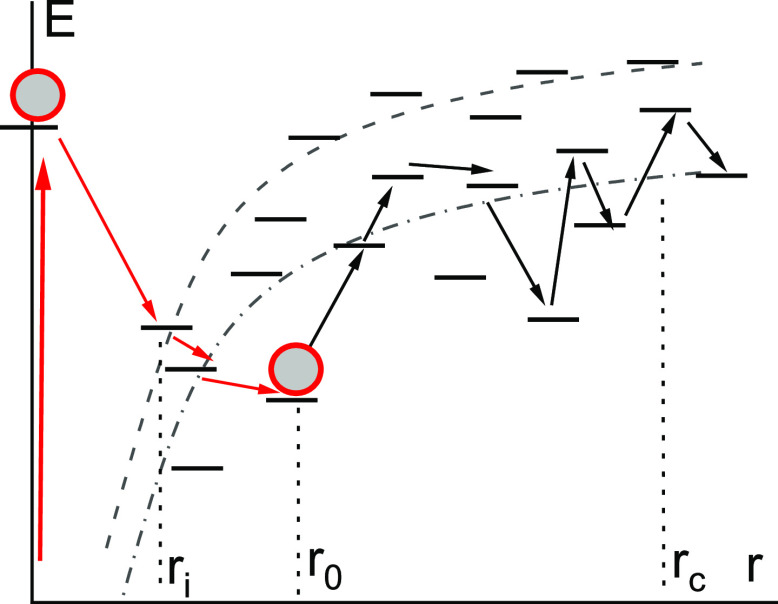
Scheme of separation of the geminate pair. The dashed and dash-dotted lines show the Coulomb interaction energy at the energy of hopping centers equal to 0 (the highest concentration of centers) and at the transport energy *Ec*, respectively.

According to the Miller-Abrahams model, the probability of hopping down in energy is not dependent on the energy of the final state. Therefore, the energy distribution of electrons is very different from that of the Fermi function. Most likely, the electron has the energy at which the density of states is maximum (this energy is further taken as the reference point, *E* = 0); i.e., the electron is “hot”. Further energy relaxation occurs in two stages. The first stage, jumps down in energy (red arrows in Figure 1), occurs until the electron falls below the energy level for which the probabilities of hopping down and up in energy are equal. This level approximately coincides with the transport level *E*_C_, which is analogous to the mobility edge.^10,18^ The first stage ends at some time *t*_s_ (the segregation time;^10,20^ the moment *t* = 0 corresponds to the exciton decay time). The second stage, *t* > *t*_s_, is a drift-diffusion motion controlled by thermal activation jumps from states with energies *E* < *Ec* to states with energies near the transport level.

According to the classical Onsager model, it is necessary to solve the Smoluchowski equation
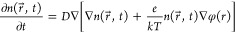
1with the boundary conditions *n*(∞,*t*) = 0, *n*(0,*t*) < ∞,^5^ to obtain the survival probability of a geminate pair until time *t*
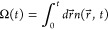
2and the separation probability, Ω_∞_ = lim_t__→∞_Ω(*t*), where *n*(*r⃗*,*t*) is the distribution function of mutual positions of charge carriers at a given time (in a typical case of great asymmetry of mobilities it is a position of the more mobile carrier (say, an electron) relative to the less mobile carrier, *D* is the diffusion coefficient, *T* is the absolute temperature, *k* is the Boltzmann’s constant, and φ(*r*) is the electrostatic potential (including the Coulomb potential)). Equation 1 is written for quasi-equilibrium transport conditions.

It should be noted that transport occurs in an anomalous regime if the initial energy distribution of charge carriers is far from quasi-equilibrium. Operationally, at any moment of time one can consider two fractions of hopping centers: the “transport states”, which make the main contribution to the transfer process, and traps, after capture in which the carrier is delayed, and apply the formalism of the multiple trapping model.^21^ In the initial time interval (after the photogeneration pulse), which may exceed the separation time of the geminate, the transport has a number of anomalous characteristics and is described by specific equations (extremely nonequilibrium, or dispersive transport).^2,10,12,18,22,23^

One can find the separation probability as a solution of a steady-state problem, assuming a stationary generation rate of carriers at a given initial position, *r⃗*_0_, and finding the normalized flux of carriers across an infinite sphere. Alternatively, one has to solve the problem in the space of initial positions with other boundary conditions.^5^ In either case, the steady-state Smoluchowski equation
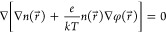
3does not depend on the diffusion coefficient. It seems (erroneously) that the separation probability does not depend on peculiarities of transport of “geminies”: whether it occurs in quasi-equilibrium or not, whether it is band-like or hopping, etc. It is important that the Smolukhovskii equation includes the thermal energy, *kT*, that results from Einstein’s ratio, *D*/μ = *kT*/*e*.

For the case of dispersive transport, the analogue of the Smoluchowski equation
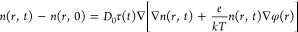
4where φ(*r*) = −*e*/4πεε_0_*r*, has been used previously in many works; see, for example.^22−24^ In eq 4*D*_0_ is the diffusion coefficient of *conductive* carriers, and τ(*t*) is an increasing function of time with the meaning of the time-dependent lifetime of conductive carriers until trapping to the “currently deep” states, i.e., those states for which the release of a previously captured carrier is unlikely until time *t*. Population of these states is far from quasi-equilibrium, but until an equilibration time, *t*_eq_, the majority of carriers occupy states below this energy, so that energy distribution is essentially “hot”, which causes the dispersive (or extremely nonequilibrium) transport regime.^2,25^ In eq 4, where *E*_d_ (*t*) is the time-dependent energy separating the “currently deep” and the “currently shallow” states,^18,19^*N*_t_ is the concentration of states.

It can be argued that the term *e*/*kT* persists in eq 4. However, an analysis shows that at low temperatures (*kT* ≪ *E*_0_, *E*_0_ is the energy scale of the density of states (DOS)) during the initial time interval, *t* ≪ *t*_s_ ≪ *t*_eq_, the kinetics of energy relaxation and transport essentially does not differ from the low-temperature limit (*T* = 0), since relaxation and transport occur by hopping down in energy. See Supporting Information, section 1. In this case, for exponential DOS, it has been shown that the energy scale of the DOS, *E*_0_, replaces the thermal energy, *kT*, in the relation between dispersion and drift shift.^10,26^ In fact, the process of initial thermalization of “hot” carriers takes place at *t* < *t*_s_, and the Onsager model can only be applied at *t* > *t*_s_, taking into account the dispersive nature of the transport, see eq 4. We use the spatial distribution of carriers at time *t*_s_, Φ(*r*_0_), as the distribution over the true initial separations *r*_0_.
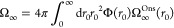
5

In eq 5 Ω_∞_^Ons^(*r*_0_) = exp(−*r*_C_/*r*_0_),^5,6^ where *r*_C_ = *e*^2^/(4πεε_0_*kT*) is the Onsager radius (Coulomb radius), and ε is the relative dielectric permittivity. In this work, one assumes a small external field strength, *F*, namely, (*eFr*_C_/*kT* ≪ 1).

In this Letter, it is shown that carrier transport in the initial energy relaxation regime can also be described (in the diffusion approximation) by the Smoluchowski equation in the dispersive (strongly nonequilibrium) transport regime; see Supporting Information, section 1. However, in this equation the drift term contains the factor *g*[*E*_d_(*t*)]/*∫*_-∞_^*E*_d_(*t*)^d*Eg*(*E*) instead of 1/*kT*, where *g*(*E*) is the DOS function, and *E*_d_(*t*) is the energy separating states populated in the quasi-equilibrium and in the nonequilibrium manner.^10,18,19,22^ The distribution Φ(*r*_0_) = *n*_i_(*r* = *r*_0_,*t*_s_) is found by solving the Smoluchowski equation in the dispersive mode of transport for the spatial-temporal distribution function of the mobile “twin”, *n*_i_ (*r*,*t*)
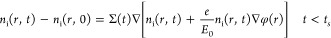
6where *n*_i_(*r*,0) = δ(*r* – *r*_i_)/(4*πr*_i_^2^), ∑(*t*) = [ln(ν_0_*t*)/(2γ)]^2^, γ is the inverse localization radius of the wave function, and ν_0_ is the frequency factor of the Miller-Abrahams model. The solution of eq 6 is obtained in the semiclassical Wentzel-Kramers-Brillouin (WKB) approximation; see Supporting Information section 3. To show the capabilities of the model over a wide temperature range, consider the exponential energy distribution of hopping centers: *g*(*E*) = (*N_t_*/*E*_0_)exp(*E*/*E*_0_), *E* < 0, where *N*_tot_ = *a*^–3^. The time interval of the dispersive mode of transport is especially wide for exponential distribution. For this DOS, *t*_s_ = ν_0_^–1^ exp(2.32*E*_0_/*kT*), see Supporting Information, section 2.

The initial energy relaxation of mobile charge carriers in a geminate pair (for definiteness, electrons) is considered. As a result, a distribution of primarily thermalized carriers arises instead of carriers separated by the initial distance. After the first jump, a geminate pair is formed, separated by *r*_i_ ≅ *a*. The formed distribution is characterized by larger values of distances *r*_0_. The quasi-equilibrium distribution is not yet established by the time *t*_s_; at *kT* ≪ *E*_0_ the dispersive mode is limited only by the filling of deep states for exponential DOS. It should be expected that the separation probability of thermalized pairs will be much greater and will depend less on temperature than what follows from the Onsager model in the case of *r*_0_ = *r*_i_.

Figure 2 shows the results of our Monte Carlo calculations of the survival probability of the geminate pair as a function of time for several temperatures, as shown in Figure 2. The simulation data are given in Supporting Information, section 4. The lattice constant *a*_0_ = *a* and localization radius are relatively large, which allow the carrier to go beyond the Onsager radius after a relatively small number of hops. This circumstance increases the significance of the initial energy relaxation (hopping down in energy). Indeed, the initial time dependence of the survival probability is universal; i.e., it does not depend on temperature.

**Figure 2:**
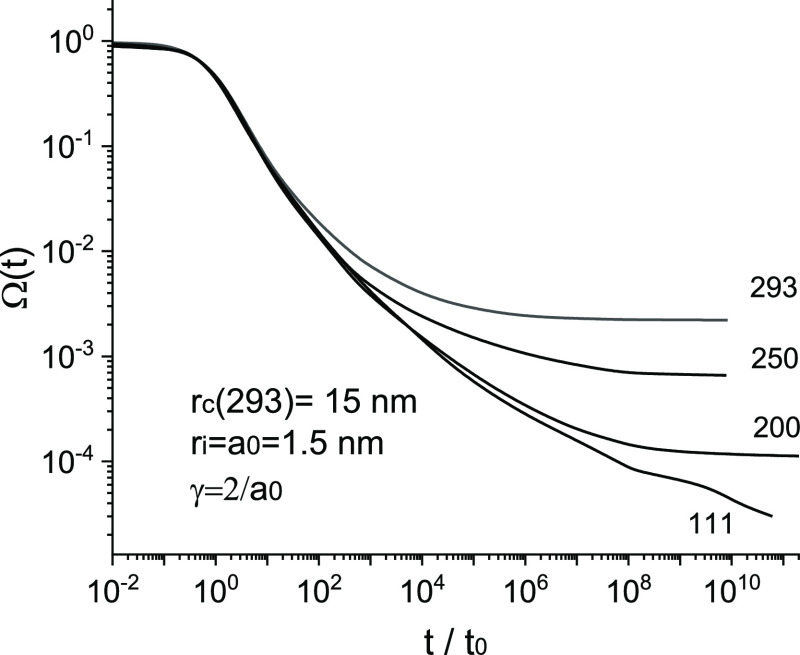
Time dependence of the survival probability of the geminate pair for several temperatures, in Kelvin, obtained from Monte Carlo simulations. Other parameters are ε = 3.8, *E*_0_ = 0.05 eV, *v*_0_ = 10^13^ s^–1^, and . *t*_0_ = exp(2*γa*_0_)/ν_0_ ≈ 5.46 × 10^−12^ s is the typical hopping time.

The results of the analytical model and Monte Carlo calculations are compared in Figures 3 and 4. Both the Monte Carlo calculations and the analytical model, see eq 5, give quantum yield values that are significantly higher than those of the Onsager model (provided that *r*_0_ = *r*_i_ = *a*_0_), see Figure 3. The analytical results are in qualitative agreement with the Monte Carlo data, showing a relatively weak dependence on temperature and a decrease in the quantum yield with a decrease in the typical hop length (the latter decreases with a decrease in the localization radius). The initial energy relaxation of charge carriers due to the energy disorder significantly increases the effective initial separation of geminate pairs. One can see that Ω_∞_ ≈ exp(−*r*_c_/*r*_0_^eff^), *r*_0_^eff^ > *r*_i_, if the temperature is not too low, since the initial (*t < t*_S_) diffusion is stronger than that at longer times. This explains why researchers often use the values *r*_0_ = *r*_0_^eff^ ≥ 2 nm for fitting experimental data, which is considerably larger than *r*_i_ ≈ *a*_0_. This effective initial separation increases with decreasing temperature, which weakens the temperature dependence of the separation probability. The results of this model are not quantitatively accurate, first, since an exponential distribution of hopping centers is considered instead of a Gaussian, which is typical for organic materials,^2^ and second, it is assumed that the characteristic energy in eq 4, replacing *kT*, is equal to *E*_0_ at *t < t*_S_ and *kT* at *t > t*_S_, although the transition between these asymptotes occurs gradually. However, it should be noted that recently, both experimental and theoretical arguments have been proposed in favor of the exponential tails of the energy distribution of hopping centers in some organic materials.^27^

**Figure 3:**
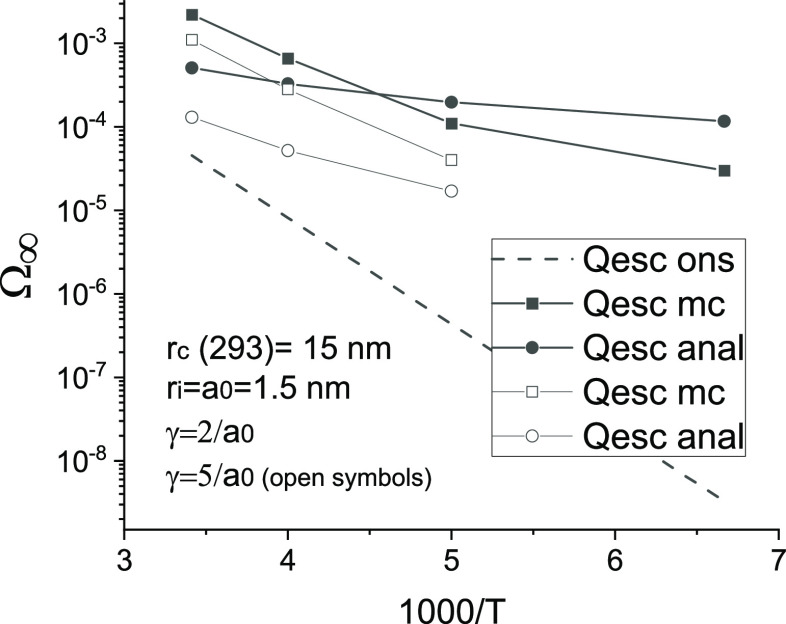
Comparison of Monte Carlo results (squares) and the analytical model (circles) for the temperature dependence of the separation probability (quantum yield) of the geminate pair. The dashed line shows the result of the Onsager model at *r*_0_ = *r*_i_ = *a*_0_. The other parameters are the same as for Figure 2.

**Figure 4:**
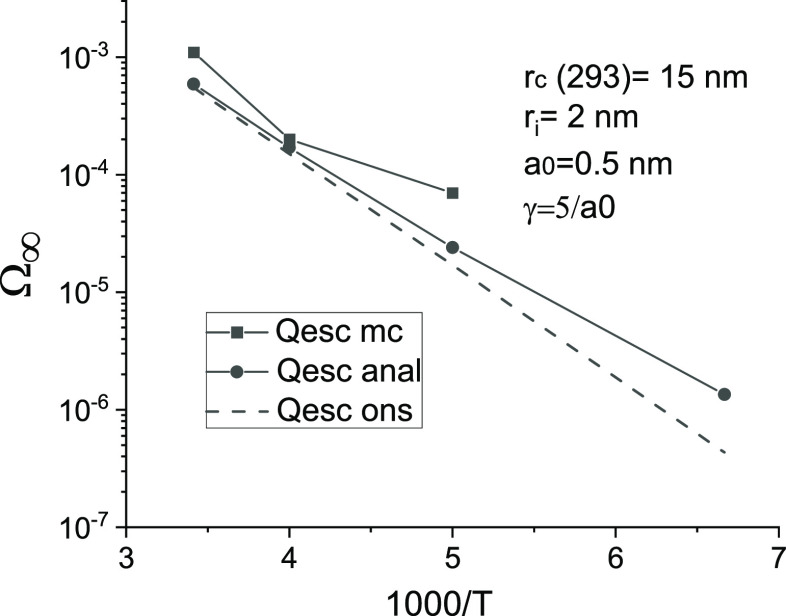
Same as in Figure 3, but in the case of a small typical jump length and strong localization. *t*_0_ = 2.2 × 10^–9^ s. The other parameters are the same as for Figure 2.

Figure 4 shows the results of test calculations that were carried out for parameter values that are more consistent with the classical Onsager model (*a*_0_ ≪ *r*_i_, strong localization). As expected, the results are relatively close to those of the Onsager model (dashed line). The Monte Carlo method gives somewhat larger values due to the finiteness of the jump length, in accordance with the known results.^13^ Thus, the analytic results stress the significant influence of the hopping parameters, namely, the localization parameter, γ*a*, and the degree of disorder, *kT*/*E*_0_, on the temperature dependence of the photogeneration quantum yield.

## Supporting Information Available

Equations of dispersive hopping transport (section 1); characteristic energies and times (section 2); solution for escape probability in WKB approximation (section 3); algorithm of Monte Carlo numerical simulation (section 4) (PDF)
