# Supplementary material for: Disorder and Photogeneration Efficiency in Organic Semiconductors
Source: J Phys Chem Lett. 2023 Aug 28;14(35):7892–6. doi: 10.1021/acs.jpclett.3c02120 (PMC10494222; doi:10.1021/acs.jpclett.3c02120)
Supplement: Supplementary file 1 — jz3c02120_si_001.pdf [file jz3c02120_si_001.pdf]

Supporting Information for

Disorder and Photogeneration Efficiency in Organic Semiconductors

Artem V. Toropin[1], Vladimir R. Nikitenko[1,*], Nikolai A. Korolev[1] and Oleg V. Prezhdo[2,*]

[1]National Research Nuclear University "MEPhI" (Moscow Engineering Physics Institute), Moscow 115409, Russia

[2]Department of Chemistry, University of Southern California, Los Angeles, CA 90089, United States

Section 1. Equations of the dispersive hopping transport.

We start from the well-known master equation for hopping transport [21],

$$\frac{\partial f_i}{\partial t} = \sum_j \nu_{ji} f_j - \nu_{ij} f_i \qquad \text{(S1)}$$

where f_i, f_j are occupation probabilities of states i and j, and ν_{ij} and ν_{ji} are rates of hopping from i to j and vice versa, as defined by the Miller-Abraham's (MA) model. Next, we will describe the localized states by their energy distribution (DOS function), $g(E)$, and by means of this DOS we rewrite the equation for the energy distribution of occupied states, $\rho(E,x,t) = g(E)f(E,x,t)$, passing from summation to integration:

$$\frac{\partial \rho(E,x,t)}{\partial t} = g(E)4\pi \int dE' \int dr r^2 \tilde{\omega}(E',E,r)\rho(E' - eFrs, x - rs, t) - \omega(E)\rho(E,x,t). \qquad \text{(S2)}$$

* Corresponding authors. Email: vladronik@yandex.ru, prezhdo@usc.edu

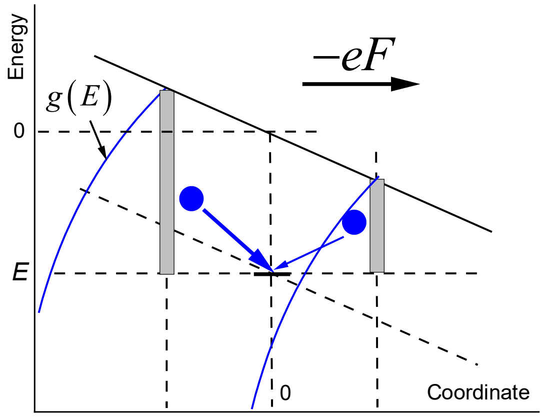

Figure S1. Scheme of electron hops in an electric field of strength *F*. The shaded rectangle shows the energy regions of the states from which hops to a state with a given energy *E* are possible at a small temperature.

Equation S2 takes into account that the electron energy includes the electrostatic energy of the external field, see fig. S1. The concentration of charge carriers is

$$N(x,t) = \int dE\, \rho(E,x,t), \tag{S3}$$

where *x* is a macroscopic co-ordinate, *r* is a (microscopic) hopping distance, *e* is elementary charge, $s = cos\theta$, θ is the angle between the direction of electric force, $e\vec{F}$, and the direction of a jump, $\vec{r}$.

The escape rate from a state with energy *E* is

$$\omega(E) = \int dE' \int d\vec{r}\, g(E)\tilde{\omega}(E,E',r). \tag{S4}$$

Unlike the hopping rates $\nu_{ij} = \nu(E,E',r) = \nu_0 exp\{-2\gamma r - [|E_j - E_i| + (E_j - E_i)]/2kT\}$ in eq. (1), the hopping rate $\tilde{\omega}(E,E',r)$ takes into account the possibility of going to states other than the final state, as well as the probability that there will be no return to the initial state, see below. One can find the escape rate $\omega(E) \equiv \nu_0 exp(-u(E))$ from the following equation [21]

$$1 = 4\pi \int_{-\infty}^{E+kTu} dE' g(E') \int_0^{r_*(E,E')} drr^2 W_{esc}\,(E,E',r)$$

$$\approx \overline{W_{esc}}(E)\frac{4\pi}{3}\int_{-\infty}^{E+kTu} dE'g(E')r_*^{\,3}(E,E'), \quad \text{(S5)}$$

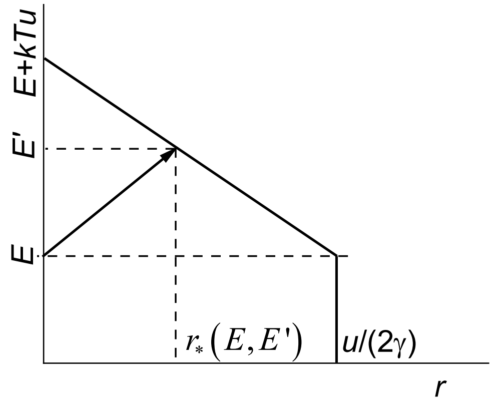

Figure S2. Region in *r-E'* space containing states for which the condition $\nu(E,E',r) > \omega(E) = \nu_0 exp(-u)$ is fulfilled.

see figure S2. According to the MA model, the hopping distance is $r_*(E',E) = (2\gamma)^{-1}\left[u(E') - \frac{E-E'}{kT}\eta(E-E')\right]$, $\eta(E'-E)$ is a step function, and $W_{esc}(E,E',r)$ is the probability that the carrier does not return to the initial state after the jump from the state of energy E to the state with energy E' over the distance r. One obtains from eq. S2, expanding the function $\rho(E'-eFrs, x-rs, t)$ in a power series in the respect to variables E' and x:

$$\frac{\partial\rho(E,x,t)}{\partial t} \approx \int dE'\widetilde{D}(E',E)\left[\frac{\partial^2\rho(E',x,t)}{\partial x^2} + 2eF\frac{\partial^2\rho(E',x,t)}{\partial x\partial E'}\right], \quad \text{(S6)}$$

where $\widetilde{D}(E',E) = \frac{4\pi}{6}\int drr^4 g(E)\widetilde{\omega}(E',E,r) \approx \frac{a^2}{6}\omega(E')$.

Next, we use the concept of time-dependent demarcation energy, below which there are "currently deep" states. The release of the carrier from these states up to the moment *t* can be neglected. Above the demarcation energy there are "currently shallow" states, from which the jump has already occurred [18]. The demarcation energy, $E_d(t)$, is defined by the condition

$$\omega[E_d(t)] \cdot t = 1 \quad \text{(S7)}$$

Integrating eq. S6 over the energy, E, and assuming that only carriers from "shallow" states can contribute to transport process, one obtains

$$\frac{\partial N(x,t)}{\partial t} = \frac{a^2(t)}{6t_0}\left[\frac{\partial^2 N_0(x,t)}{\partial x^2} + 2eF\int_{E_d(t)}^{\infty} dE' \ \omega(E')\frac{\partial^2 \rho(E',x,t)}{\partial x \partial E'}\right], \tag{S8}$$

where

$$N_0(x,t) = t_0 \int_{E_d(t)}^{\infty} dE' \omega(E')\rho(E',x,t) \tag{S9}$$

has a meaning of concentration of the most mobile fraction of charge carriers, accepting the criterion that the waiting time for a jump is no longer than t_0 [21].

After integrating by parts in eq. S8, one obtains

$$\frac{\partial N(x,t)}{\partial t} = \frac{a^2(t)}{6t_0}\left\{\frac{\partial^2 N_0(x,t)}{\partial x^2} - 2eF\frac{\partial}{\partial x}\left[t_0\omega(E_d)\rho(E_d,x,t) + t_0\int_{E_d(t)}^{\infty} dE'\rho(E',x,t)\frac{d\omega(E')}{dE'}\right]\right\} \tag{S10}$$

Below in this section, we will consider the limiting cases of eq. S10 for small and large times, find the relationship between the functions $N_0(x,t)$ and $N(x,t)$, and obtain an equation for the function $N(x,t)$ from eq. S10 in the limiting cases.

One can write a non-equilibrium energy distribution, $\rho(E,x,t)$, in the following separable form, considering that 1) the length scale of variations with x considerably exceeds the hopping length; 2) $\rho(E)$ follows the Boltzmann's function, if $E \gg E_d(t)$; 3) $\rho(E)$ is a constant, if $E \ll E_d(t)$, because the rate of downward jumps does not depend on the final energy:

$$\rho(E,x,t) = g(E)f(E,x,t) = \frac{g(E)}{1+exp\left[\frac{E-E_d(t)}{kT}\right]}f_0(x,t). \tag{S11}$$

If temperature is small, eq. S11 approaches to the step function,

$$\rho(E,x,t) \approx \frac{g(E)N(x,t)}{G[E_d(t)]}\eta[E_d(t) - E], \ kT \ll E_0 \tag{S12}$$

In eq. S11, $f_0(x,t) \approx N(x,t)/G[E_d(t)]$ is the low-energy limit of occupation probability, where $G(E) = \int_{-\infty}^{E} dE\, g(E)$. One obtains from eqs. S11, S12,

$$\rho(E_d,x,t) = \frac{1}{2}g(E_d)f_0(x,t) = \frac{g(E_d)N(x,t)}{2G[E_d(t)]}. \tag{S13}$$

In order to establish the relationship between the functions $N(x,t)$ and $N_0(x,t)$, we consider eq. S2, neglecting the spatial non-uniformity of the function ρ on the scale of a typical hopping distance, a, and assuming a small electric field strength, F. Eq. S2 reduces to

$$\frac{\partial \rho(E,x,t)}{\partial t} = g(E) 4\pi \int dE' \int dr r^2 \widetilde{\omega}(E',E,r)\rho(E',x,t) - \omega(E)\rho(E,x,t) \tag{S14}$$

Considering the "currently deep" states (neglecting the release term in eq. S14), and considering the hopping rate $\widetilde{\omega}(E,E',r)$ as

$$\widetilde{\omega}(E',E,r) = \omega(E')W_{esc}(E',E,r)\eta(r_*(E',E) - r),$$

one obtains

$$\frac{\partial \rho(E,x,t)}{\partial t} = g(E)\frac{4\pi}{3}\int_{E_d(t)}^{\infty} dE' \omega(E')\overline{W_{esc}}(E') r_*{}^3(E')\, \rho(E',x,t),\ E < E_d(t) \tag{S15}$$

Since preferably the jumps occurs over the distance $r = r_*$, see fig S2, and typically the jumps to the "currently deep" states occur downwards in energy, $r_* = r_*(E') = u(E')(2\gamma)^{-1}$. Expressing the concentration of "mobile" carriers from eqs. S9, S11,

$$N_0(x,t) = t_0 f_0(x,t) \int_{E_d(t)}^{\infty} dE \frac{g(E)\omega(E)}{1+exp\left[\frac{E-E_d(t)}{kT}\right]}, \tag{S16}$$

one can rewrite eq. S15 as follows,

$$\frac{\partial \rho(E,x,t)}{\partial t} = g(E)\frac{\frac{4\pi}{3}\int_{E_d(t)}^{\infty} dE' g(E')\omega(E')\overline{W_{esc}}(E') r_*{}^3(E')\left[1+exp\left[\frac{E-E_d(t)}{kT}\right]\right]^{-1}}{t_0 \int_{E_d(t)}^{\infty} dE' g(E)\omega(E')\left[1+exp\left[\frac{E-E_d(t)}{kT}\right]\right]^{-1}} N_0(x,t) \tag{S17}$$

Integrating this equation over time and energy E from $-\infty$ to $E_d(t)$, under the reasonable assumption that majority of carriers occupy the currently deep states ($kT \ll E_0, t \ll t_{eq}$), one obtains the usual for the dispersive transport regime [18, 22] relationship between the functions $N(x,t)$ and $N_0(x,t)$,

$$N(x,t) = \tau^{-1}(t)\int_0^t dt' N_0(x,t'), \tag{S18a}$$

$$\text{i. e. } N_0(x,t) = \frac{\partial}{\partial t}[\tau(t)N(x,t)], \tag{S18b}$$

where

$$\tau(t) = t_0\{\Omega(t)G[E_d(t)]\}^{-1}, \tag{S19}$$

$$\Omega(t) = \frac{\frac{4\pi}{3}\int_{E_d(t)}^{\infty} dE' g(E')\omega(E')\overline{W_{esc}}(E') r_*^{\,3}(E')\left[1+exp\left[\frac{E-E_d(t)}{kT}\right]\right]^{-1}}{\int_{E_d(t)}^{\infty} dE' g(E)\omega(E')\left[1+exp\left[\frac{E-E_d(t)}{kT}\right]\right]^{-1}}, \quad \text{(S20)}$$

Since the population of the "currently deep" states occurs preferably by downward jumps (in energy), $r_*(E', E) = r_*(E') = (2\gamma)^{-1} ln[\nu_0/\omega(E')]$ in eq. S20. On the other hand, one can rewrite eq. S15 in the form

$$\frac{\partial f_0(x,t)}{\partial t} = \frac{4\pi}{3}\int_{E_d(t)}^{\infty} dE' g(E')\omega(E')\overline{W_{esc}}(E') r_*^{\,3}(E')\left[1+exp\left[\frac{E-E_d(t)}{kT}\right]\right]^{-1} f_0(x,t) \quad \text{(S21)}$$

For the case of a long time, $t \gg t_s$, where the time t_s is defined by the condition $E_d(t_s) = E_{1/2}$, and $E_{1/2}$ is the energy of equal probabilities of downward and upward (in energy) jumps, see section 2, the following approximations are valid: $E_d(t) \ll E_{1/2}$, $E_d(t) \approx E_{tr} - kTln(\nu_0 t)$, $\omega(E') \approx \nu_0 \exp[-(E_{tr} - E')/kT]$, if $E' < E_{1/2}$ (E_{tr} is an *effective* (formal) transport energy, a different value than $E_{1/2}$, see section S2), and $\overline{W_{esc}}(E) \equiv B^{-1}(E) \approx B_0^{\,-1}, B_0 \approx 2.77$ [17, 19], hence in eq. S21

$$\omega(E')\left[1+exp\left[\frac{E-E_d(t)}{kT}\right]\right]^{-1} \approx \nu_0 exp[-(E_{tr} - E_d)/kT] = \frac{1}{t} = \omega[E_d(t)],$$

$r_*(E') = (2\gamma)^{-1}[(E_{tr} - E')/kT]^3$, and eq. S21 reduces to

$$\frac{\partial f_0(x,t)}{\partial t} = \frac{1}{t}\left[\frac{4\pi}{3} B_0^{\,-1}\int_{-\infty}^{E_{tr}} dE' g(E') r_*^{\,3}(E')\right] f_0(x,t) = f_0(x,t)/t. \quad \text{(S22)}$$

Using eqs. S13 and S22, one can express the first term in the square brackets in eq. S10 as follows,

$$t_0\omega[E_d(t)]\rho(E_d, x, t) = \frac{t_0}{2} g(E_d)\frac{f_0(x,t)}{t} = \frac{t_0}{2} g(E_d)\frac{\partial f_0(x,t)}{\partial t} = \frac{t_0}{2}\frac{g(E_d)}{G(E_d)}\frac{\partial N(x,t)}{\partial t} \quad \text{(S23)}$$

One has to note that the energy $E_{1/2}$ plays the role of a *real* (not effective) transport level, mostly contributing to transport, if $t \gg t_s$ [18, 19].

In the same way, and replacing $E_d(t) \to -\infty$, one can also estimate the integrals in eq. S20 as follows:

$$\Omega(t) \approx \frac{\frac{4\pi}{3}\int_{-\infty}^{E_{1/2}} dE' g(E') B_0^{\,-1}(2\gamma)^{-1}[(E_{tr} - E')/kT]^3 + kTg(E_d)B_0^{\,-1}}{\int_{-\infty}^{E_{1/2}} dE' g(E') + kTg(E_d)} \approx G(E_{1/2})^{-1}. \quad \text{(S24)}$$

Then, eqs. S19, S24 give

$$\tau^{-1}(t) = t_0^{-1}\frac{G[E_d(t)]}{G(E_{1/2})} = t_0^{-1}\int_{-\infty}^{E_d(t)} dE\,\frac{g(E)}{N_t}\,, \tag{S25}$$

where $N_t \equiv G(E_{1/2})$ is the concentration of "traps", i. e., states below the transport energy $E_{1/2}$, that is an analog of the mobility edge of the MT model. Eq. S25 is the same as in the previous works [18,19,22]. Considering the second term in the square brackets in eq. S10, we can rewrite it as follows:

$$2eFt_0\int_{E_d(t)}^{\infty} dE'\rho(E',x,t)\frac{d\omega(E')}{dE'} = 2eF\left\{\frac{\frac{4\pi}{3}\int_{E_d(t)}^{\infty} dE' g(E')(d\omega(E')/dE')\left[1+exp\left[\frac{E'-E_d(t)}{kT}\right]\right]^{-1}}{\int_{E_d(t)}^{\infty} dE' g(E)\omega(E')\left[1+exp\left[\frac{E'-E_d(t)}{kT}\right]\right]^{-1}}\right\}N_0(x,t)$$

$$\equiv \frac{eF}{E_2}\frac{\partial}{\partial t}[\tau(t)N(x,t)], \tag{S26}$$

where $\omega(E') \approx \nu_0 \exp[-(E_{tr}-E')/kT] \equiv \omega_0 exp[-(E_{1/2}-E')/kT]$, $E' \ll E_{1/2}$, and $\omega(E') \approx \omega_0$, $E' \gg E_{1/2}$. The states near $E_{1/2}$ give the main contribution to hopping at $t \gg t_s$, hence one can estimate the energy E_2 in eq. S26 as *kT*.

Integrating eq. S10 over time from 0 to *t* and using eqs. S18, S23, S26, one obtains

$$N(x,t) - N(x,0) = \frac{a^2}{6t_0}\tau(t)\left\{\frac{\partial^2 N(x,t)}{\partial x^2} - eF\frac{g(E_d)}{G(E_d)}\frac{t_0}{\tau(t)}\frac{\partial N(x,t)}{\partial x} - \frac{eF}{kT}\frac{\partial N(x,t)}{\partial x}\right\}, \tag{S27}$$

where one can estimate $a \approx (2\gamma)^{-1}ln(\nu_0 t_s)$. Since $\tau(t)$ increases with time, the second term in the curly brackets is small in comparison with the 3^{d} term, and one obtains

$$N(x,t) - N(x,0) = \frac{a^2}{6t_0}\tau(t)\left\{\frac{\partial^2 N(x,t)}{\partial x^2} - \frac{eF}{kT}\frac{\partial N(x,t)}{\partial x}\right\},\ t \gg t_s \tag{S28}$$

Thus, the Einstein relation is fulfilled, if the time is rather long, $t \gg t_s$., i. e., transport is controlled by upward jumps from "currently shallow" (i. e. in quasi-equilibrium) states to the states near the transport level, that contributes mostly to the transport [18].

For the opposite case, $t \ll t_s$, $E_d(t) \gg E_{1/2}$, release from the "currently shallow" states by hopping downwards in energy prevails, and these states are located near the demarcation energy, $E_d(t)$. Hence, the typical release time is *t*, and $B^{-1}(E) \approx 1$ (return to initial state is not probable). From eq. S5 one obtains $G(E)\frac{4\pi}{3}r_*(E)^3 = 1$, $r_*(E) = [(4\pi/3)G(E)]^{-1/3}$, $\omega(E) = \nu_0 exp(-2\gamma r_*(E)) \approx \nu_0 exp(-2\gamma r_*(t)) = 1/t$, $r_*(t) \equiv r_*[E_d(t)] = (2\gamma)^{-1}ln(\nu_0 t)$, $t \ll t_s$. One can estimate eq. S15 as,

$$\frac{\partial\rho(E,x,t)}{\partial t} = \frac{g(E)}{t_0}\frac{N_0(x,t)}{G(E_d)},\ \ E < E_d(t) \tag{S29}$$

Integrating eq. S29 over energy E from $-\infty$ to $E_d(t)$, and subsequently over time from 0 to t, one obtains

$$\frac{\partial N(x,t)}{\partial t} = \frac{N_0(x,t)}{t_0}, N(x,t) = \int_0^t dt'\, N_0(x,t')/t_0 \tag{S30}$$

On the other hand, considering downward jumps at the short time domain, $t \ll t_s$, and considering that the jumps occur from the narrow region near the energy $E_d(t)$, one obtains

$$\frac{\partial \rho(E,x,t)}{\partial t} = g(E)\int_E^\infty dE' \frac{\omega(E')}{G(E')}\rho(E',x,t) \approx \frac{g(E)}{G(E_d)}\frac{1}{t}\int_{-\infty}^\infty dE' \rho(E',x,t) = \frac{g(E)}{G(E_d)}\frac{N(x,t)}{t}$$

After integrating over energy, one obtains

$$\frac{\partial N(x,t)}{\partial t} = \frac{N(x,t)}{t}$$

Since $f_0(x,t) \approx N(x,t)/G\{E_d(t)\}$, one obtains

$$g(E_d)\omega[E_d(t)]f_0(x,t) \approx \frac{g(E_d)}{G(E_d)}\frac{\partial N(x,t)}{\partial t} \tag{S31}$$

Estimating the field-assisted term in eq. S10 in the short-time domain, $t \ll t_s$, one obtains

$$-\frac{eFt_0 g(E_d) f_0(x,t)}{t}\left[1 + \frac{\partial\omega(E_d)/\partial E}{\omega(E_d)}\int_{E_d}^\infty dE'\, exp\left(-\frac{E'-E_d}{kT}\right)\right], \text{ i.e., } -\frac{eFt_0 g(E_d) f_0(x,t)}{t}\left[1 + \frac{2ln(\nu_0 t)}{3}kT\frac{g(E_d)}{G(E_d)}\right]$$

The second term is small for the relevant case $kT/E_0 \ll 1.$, and the first term is, see eq. S31,

$$-\frac{eFt_0 g(E_d)}{G(E_d)}\frac{\partial N(x,t)}{\partial t}$$

Neglecting the second term, integrating eq. S10 over time and using eqs. S30, S31, one obtains the temperature-independent drift-diffusion equation in the dispersive transport regime:

$$N(x,t) - N(x,0) = \frac{a^2(t)}{6}\left\{\frac{\partial^2 N(x,t)}{\partial x^2} - \frac{g(E_d)}{G(E_d)}\frac{\partial N(x,t)}{\partial x}\right\}, \underline{t \ll t_s}. \tag{S32}$$

One can argue that both eqs. S28 and S32 do not depend on the value of the time t_0, the same as in the quasi-equilibrium regime [21]. Comparing eqs. S28 and S32, one can argue that the thermal energy kT is replaced in eq. S32 by the temperature-independent term, $\frac{g(E_d)}{G(E_d)}$. For the case of exponential DOS, $G(E_d)/g(E_d) = E_0 = const.$ In eqs. S28 and S32,

$$a^2(t) = r_*^2(t) = (2\gamma)^{-1}ln(\nu_0 t), t \ll t_s;\ a^2 = (2\gamma)^{-1}ln(\nu_0 t_s), t \gg t_s. \tag{S33}$$

Section 2. Characteristic energies and times.

The energy $E_{1/2}$ is defined by the condition

$$n_{\uparrow}(E_{1/2}) = n_{\downarrow}(E_{1/2}), \tag{S34}$$

where $n_{\uparrow}$ and $n_{\downarrow}$ are the numbers of available neighbor states upwards and downwards in energy for the given escape rate, $\omega = \exp(-u)$,

$$n_{\downarrow} = \frac{4\pi}{3}\left(\frac{u}{2\gamma}\right)^3 \int_{-\infty}^{E} dE' g(E'),$$

$$n_{\uparrow} = \frac{4\pi}{3}\int_{E}^{E+kTu} dE' g(E')\left(\frac{E+kTu-E'}{2\gamma kT}\right)^3$$

The energy $E_{1/2}$ and the respective hopping parameter, u_s, are related as

$$\int_{E_{1/2}}^{E_{1/2}+kTu_s} dE'\, g(E')\left(\frac{E+kTu_s-E'}{2\gamma kT}\right)^3 = \left(\frac{u_s}{2\gamma}\right)^3 \int_{-\infty}^{E_{1/2}} dE'\, g(E'). \tag{S35}$$

For the case of an exponential energy distribution of hopping centers,

$$g(E) = \frac{N_{tot}}{E_0} exp\left(\frac{E}{E_0}\right), E < 0, \tag{S36}$$

one obtains from eq. S35 $u_s \approx 2.32E_0/kT$. Hence, the downward jumps regime occurs until the time

$$t_s = {\nu_0}^{-1} exp(2.32E_0/kT) \tag{S37}$$

Assuming a sufficiently deep initial state, $E \to -\infty$, neglecting downward jumps for this case, and introducing the energy $E_{tr} = E + kTu$, one can reduce eq. S5 to

$$\frac{4\pi}{3}\int_{-\infty}^{E_{tr}} dE'\, g(E')\left(\frac{E_{tr}-E'}{2\gamma kT}\right)^3 = B_0, \tag{S38}$$

$B_0 \approx 2.77$ [19]. The energy E_{tr} does not depend on the initial energy E. Hence, the escape rate from the deep tail of the DOS is

$$\omega = \nu_0 \exp\left(-\frac{E_{tr}-E}{kT}\right). \tag{S39}$$

Eq 39 is reminiscent to thermal activation from energy E to energy E_{tr} with a zero hopping length. Hence, the energy E_{tr} (the effective, or formal transport level) [18] is the upper limit for the available energies. It gives the expression for the release rate from deep states in the most compact way.

Section 3. Solution for the escape probability in WKB-approximation

On the basis of results of Section 1 above, we can write a Smoluchowski equation for the dispersive transport regime of "geminies",

$$n(\vec{r},t) - n(\vec{r},0) = D_0\tau(t)\left[\nabla^2 n(\vec{r},t) + \frac{1}{E_*(t)}\nabla n(\vec{r},t)\nabla\varphi(\vec{r})\right], \tag{S40}$$

where $D_0 = a^2/6t_0$, see eq 33. For the case of a negligible external field (the case of central symmetry), $\varphi(r) = -e/4\pi\varepsilon\varepsilon_0 r$, eq. S40 gives

$$\frac{D_0\tau(t)}{r^2}\frac{\partial}{\partial r}\left[r^2\frac{\partial n}{\partial r} + \frac{e^2}{4\pi\varepsilon\varepsilon_0 E_*(t)}n(r,t)\right] - n(r,t) = -\frac{\delta(r-r_i)}{4\pi r_i^2}, \tag{S41}$$

provided that $n(r,0) = \delta(r-r_i)/4\pi r_i^2$. Eq. S41 is an ordinary differential equation. It includes time as a parameter. Introducing dimensionless variables and parameters, $\tilde{n} = n r_c^3$, $x = r/r_c$, $x_i = r_i/r_c$, $r_c = e^2/4\pi\varepsilon\varepsilon_0 kT$ (the Coulomb radius), $O^2(t) = r_c^2/D_0\tau(t)$, $\xi(t) = E_*(t)/kT$, we can rewrite eq. S41 as

$$\frac{d^2\tilde{n}}{dx^2} + \frac{d\tilde{n}}{dx}\left(\frac{2}{x} + \frac{1}{\xi(t)x^2}\right) - O^2(t)\tilde{n}(r,t) = -O^2(t)\frac{\delta(x-x_i)}{4\pi x_i^2} \quad .. \tag{S42}$$

Below, we use the following approximation for the function $E_*(t)$ (combination of asymptotes):

$$E_*(t) = E_0, t \le t_s;\ E_*(t) = kT,\ t > t_s. \tag{S43}$$

Replacing the function in eq. S43,

$$\tilde{n} = \frac{O^2(t)}{4\pi x_i x}\exp\left[\frac{1}{2\xi x} - \frac{1}{2\xi x_i}\right]z(x,t), \tag{S44}$$

one obtains the following equation

$$\frac{d^2 z}{dx^2} - \kappa^2(x,t)z(x,t) = -\delta(x-x_i), \tag{S45}$$

$$\kappa(x,t) = \left[\frac{1}{4\xi^2 x^4} + O^2(t)\right]^{1/2} \tag{S46}$$

Integrating eq. S42 over an infinitely thin interval around x_i, one obtains the boundary conditions

$$z(x_i+0,t) = z(x_i-0,t), \quad \frac{dz(x_i+0,t)}{dx} - \frac{dz(x_i-0,t)}{dx} = -1 \tag{S47}$$

Other boundary conditions result from the requirement that function $\tilde{n}$ is finite at x=0 and approaches zero as x approaches infinity.

We find the solution of eq. S45 in the WKB approximation with the boundary conditions S47, and eq. S44

$$\tilde{n}\,(x,t) = \frac{O^2(t)\exp\left(\frac{1}{2\xi x}-\frac{1}{2\xi x_i}\right)}{8\pi x_i x\sqrt{k(x,t)}\sqrt{k(x_i,t)}}\begin{cases}\exp\left(-\int_x^{x_i}\kappa(x',t)dx'\right), x \le x_i\\ \exp\left(-\int_{x_i}^{x}\kappa(x'',t)dx'\right), x > x_i\end{cases} \tag{S48}$$

We can find integrals in eq. S48 in terms of hypergeometric functions,

$$\int_x^{x_i} k(x',t)dx' = \frac{x_i\ \ {}_2F_1\left(-\frac{1}{2};-\frac{1}{4};\frac{3}{4};-4x^4\xi^2 O^2(\mathrm{t})\right)-x\ \ {}_2F_1\left(-\frac{1}{2};-\frac{1}{4};\frac{3}{4};-4x_i{}^4\xi^2 O^2(\mathrm{t})\right)}{2xx_i\xi}. \tag{S49}$$

Provided that $\xi = E_0/kT$, $\tau(t) = t_0$, see eq. S32, one obtains $\Phi(r_0) \equiv n(r = r_0, t_s)$ from eqs. S48, S49, S33, and one obtains the escape probability from eq. 5 of the main text.

Section 4. Numerical Monte-Carlo simulation

At the initial moment of time, $t = 0$, at a distance r_i from a stationary hole with Cartesian coordinates (0, 0, 0), an electron is born. Further movement of the electron occurs by hopping over the nodes of the cubic lattice (a_0 is the lattice constant) with integer coordinates (m, n, k). The energy of the ith node, E_i, is set randomly according to the exponential distribution. The difference in the electron's energies when jumping from node i to node j is the sum of the differences in the Coulomb energies, random energies of nodes E_i and E_j, and the energy of a uniform electric field F:

$$\Delta U_{ij} = U_j - U_i = -\frac{e^2}{4\pi\varepsilon\varepsilon_0}\left(\frac{1}{|\vec{r}_j|}-\frac{1}{|\vec{r}_i|}\right) + E_j - E_i + e\vec{F}(\vec{r}_j - \vec{r}_i), \tag{S50}$$

where $\vec{r}_i$, $\vec{r}_j$ are radius-vectors of an electron at the nodes i and j, $|\vec{r}_i| = a_0\sqrt{m_i^2 + n_i^2 + k_i^2}$. The probability to jump between nodes i and j is $P_{ij} = \nu_{ij}/\nu_i$, $\nu_i = \sum_{l\neq i}\nu_{il}$, where ν_{ij} is calculated according to the Miller-Abrahams model:

$$\nu_{ij} = \nu_0\exp\left(-2\gamma|\vec{r}_i - \vec{r}_j| - \frac{\Delta U_{ij}+|\Delta U_{ij}|}{2kT}\right), \tag{S51}$$

The probability for an electron to stay at a given site decreases exponentially with time, according to the Poisson distribution. Therefore, the lifetime of an electron at site i before hopping to site j is $t_i = -\ln(x_0^1)\nu_i{}^{-1}$, where x_0^1 is a random number distributed uniformly on the interval (0,1).

An electron can jump to nodes which are located in a volume of 15×15×15 nodes, i. e., 3374 neighboring nodes are taken into account. The node j to which the jump will occur is determined based on the probabilities P_{ij} using random numbers. Calculations stop either when an electron arrives at a site (0, 0, 0), which is considered a recombination of a pair, or if an electron reaches distance $5r_c$ from this site, which is considered a separation of a pair. The moment of recombination is calculated by summing the times of individual hops, t_i. The calculations are repeated from 100,000 to 800,000 times, depending on temperature and other parameters, to ensure that the survival probability practically ceases to decrease with time. The survival probability is calculated as the ratio of the number of pairs that have survived to a given moment of time to the total number of born pairs.
